# Editorials

**Published:** 1902-04

**Authors:** 


### EDITORIALS.

### A QUARTER OF A CENTURY'S USEFULNESS.

Our esteemed contemporary the *American Veterinary Review* has turned the twenty-fifth milestone of its existence in the journalistic pathway of veterinary medicine.

Given birth by the U. S. V. M. A., it served for many years as the chronicle of the work of the members of that organization, and accomplished much in attracting attention to the strength and work of many of the individual members of the national organization.

The first twenty years of its career it was most ably edited by that great teacher, writer, and supporter of college and association power, A. Liautard, who, in maintaining a higher curriculum for our schools, a broader field of journalistic work, added much at all times to the worth and scope of American veterinary literature.

Like many other scientific journals, it has never had the support of the veterinary profession that it deserved, and its editing and publication have been a matter of loving devotion to an adopted profession.

The high ethical standard established by its founder and chief editor for more than a score of years did much indeed to give standing and recognition to the birth and growth of a new profession in our country.

We wish for its present editorial direction and management the same grand results in the present cycle of years; a better financial support than it has ever had before; that it may not yield to any departure from the strong ethical lines it has well advocated, but in greater returns make broader and richer its field of usefulness in adding strength and value to future American veterinary literature.

### COLUMELLA'S PROPOSITION.

The continued interest shown in "Columella's" proposition for a solution of the commercial side of tuberculosis among the bovine species continues, and it is hoped that some *State* may inaugurate the plan suggested. Perhaps this the "Keystone State" might consider the feasibility of this plan, and we most earnestly recommend to the Pennsylvania State Live-stock Sanitary Board the proposition as well as the consideration of the various views expressed by those who have contributed to its consideration. It is important, and from it may develop some cattle insurance plan that might well grow under State supervision and direction.

---

### OHIO'S SPLENDID TRIBUTE TO OUR LATE EDITOR, DR. RUSH SHIPPEN HUIDEKOPER.

The Ohio Veterinary Medical Association feels, in common with all veterinarians throughout the United States, a desire to pay its tribute of respect to the memory of the late Dr. Rush Shippen Huidekoper, who was not alone a leader in thought, but also a foremost leader in action for the cause of the science to which he had turned out of pure love of the brute creation, and out of a sincere desire to alleviate the sufferings of the dumb friends of man.

To such a supremely elevated character, adorned as he was with all that the advanced thought and skill of scientific research and expert knowledge could confer, both as regards the general practice of medicine and surgery and the equally beneficent practice of animal pathology, mere words fail to convey a full appreciation of the measured thought of his value to the world, to the profession, and to his family, and especially to us as an organization working to dignify and elevate the profession which he so richly endowed by means of his vast knowledge and his sturdy activities.

Had Dr. Huidekoper done nothing more than what he accomplished in his splendid struggle for Congressional recognition of the veterinary profession in its relation to the military arm of the government, veterinarians everywhere would owe him a debt of grateful remembrance which no language could express; but he did far more than that, as we of the inner circle can testify, and therefore it is that we thus make known our desire to place upon the records of our Association this permanent tribute to his memory, and to

*Resolve*, That in the death of Dr. Huidekoper the veterinarians of America have lost a noble champion of their cause, both in military and civil life, and the profession will feel keenly the loss of one of its greatest exponents and practitioners; and it is further

*Resolved*, That a copy of this memorial be spread on the minutes of this Association, and also that copies be sent to the various veterinary journals for publication.

William R. Howe,
L. W. Carl,
W. J. Torrance,
Committee.

William H. Gribble,
Secretary.

### OHIO'S TRIBUTE TO THE MEMORY OF THE LATE DR. A. W. CLEMENT.

At a meeting of the Ohio State Veterinary Medical Association, held at Columbus, Ohio, January 14 and 15, 1902, the following resolutions were adopted :

*Whereas*, We learn with regret of the recent death of Dr. Albert W. Clement, of Baltimore, Md., a fellow-practitioner who, by reason of the successful manner in which he has practised in his chosen profession for so many years, has established himself in our memories, and whom we wish to venerate as a man and as a fellow-practitioner, and,

*Whereas*, It has pleased the Omnipotent God to take from our profession such a noble, earnest, and valuable member, and,

*Whereas*, The Ohio State Veterinary Medical Association deeply deplores the loss of such a highly educated man, yet we feel that it is our duty to submit to the manifestations of wisdom of the Almighty God, and we therefore

*Resolve*, That the sincere sympathy of this Association be extended to his bereaved widow and family, and

*Resolve*, That a copy of these resolutions be sent to his widow, that a copy be spread on the books of this Association, and that copies be sent to the various veterinary journals for publication.

William R. Howe,
L. W. Carl,
W. J. Torrance,
Committee.

William H. Gribble,
Secretary.

